# Supplementary material for: A Biosocial Perspective to Understand Antimicrobial Prescription Practices: A Retrospective Cross-Sectional Study from a Public Community Health Center in North India
Source: Antibiotics (Basel). 2025 Feb 20;14(3):213. doi: 10.3390/antibiotics14030213 (PMC11939266; doi:10.3390/antibiotics14030213)
Supplement: Supplementary file 1 [file antibiotics-14-00213-s001.zip › antibiotics-3472754-supplementary.pdf]

Supplementary data:

Prescription Date	Month	Year	OPD	Owner of slip	Patient age	Age group	Patient sex	Location of the patient	Season	Symptom 1	Symptom 2	Symptom 3	Confirmed Diagnosis	Provisional diagnosis	Sign/ Symptom Duration (Days)	Other symptoms	Antibiotics prescribed	Multiple antibiotics prescribed	Branded/Generic	Antibiotic prescribed 1	AWaRe classification	Class of antibiot
2023-02-01	January	2023	Gynaecology	Self	31	16 to 39	Female	Rural	Winter	Abdominal pain							Yes	No	Generic	Ofloxacin + Ornidazole	Watch	Second generation Fluoro
2023-02-01	January	2023	eneral Medici	Self	60	40 to 65	Female	Rural	Winter				URC				Yes	No	Generic	Doxycycline	Access	Tetracyclines
2023-02-01	January	2023	Paediatrics	Other	1	1 to 5	Female	Rural	Winter	Fever	ed frequency of micturition		UTI		11		Yes	No	Generic	Cefixime	Watch	Third generation Cephal
2023-02-01	January	2023	Dental	Self	22	16 to 39	Female	Rural	Winter				Pericoronitis				Yes	No	Generic	Ofloxacin + Ornidazole	Watch	Second generation Fluoro
2023-02-01	January	2023	Dermatology	Other	1	1 to 5	Female	Rural	Winter				Folliculitis			Recurrent LAP	Yes	No	Generic	Amoxyclav	Access	Beta-lactam/Penicil
2023-02-01	January	2023	Paediatrics	Other	8	6 to 15	Male	Urban	Winter	Fever			Tonsillitis		2		Yes	No	Generic	Cefuroxime	Watch	Second generation Cepha
2023-06-01	January	2023	Gynaecology	Self	29	16 to 39	Female	Rural	Winter	Painful/ Burning micturition	White discharge				3		Yes	No	Generic	Nitrofurantoin	Access	Nitrofurans
2023-06-01	January	2023	Dermatology	Self	35	16 to 39	Male	Rural	Winter	Trauma			Cellulitis				Yes	No	Generic	Cefixime	Watch	Third generation Cephal
2023-09-01	January	2023	Paediatrics	Other	1	1 to 5	Male	Urban	Winter				URC	UTI??			Yes	No	Generic	Cefpodoxime	Watch	Third generation Cephal
2023-09-01	January	2023	Dental	Self	20	16 to 39	Female	Rural	Winter	Tooth pain					7		Yes	No	Generic	Ofloxacin + Ornidazole	Watch	Second generation Fluoro
2023-09-01	January	2023	Paediatrics	Other	5	1 to 5	Female	Rural	Winter	Fever	Cold/ Runny nose	ough (productive	ARI		14		Yes	No	Generic	Cefpodoxime	Watch	Third generation Cephal
2023-09-01	January	2023	Anasthesia	Self	35	16 to 39	Female	Rural	Winter	Fever	Sore throat				1		Yes	No	Generic	Cefixime	Watch	Third generation Cephal
2023-09-01	January	2023	eneral Medici	Self	34	16 to 39	Female	Urban	Winter				Tonsillitis				Yes	No	Generic	Amoxyclav	Access	Beta-lactam/Penicil
2023-09-01	January	2023	Dermatology	Self	50	40 to 65	Female	Rural	Winter	Boil in forehead							Yes	No	Generic	Amoxyclav	Access	Beta-lactam/Penicil
2023-09-01	January	2023	Paediatrics	Other	4	1 to 5	Male	Rural	Winter	Abdominal pain			URC	UTI??			Yes	No	Generic	Cefpodoxime	Watch	Third generation Cephal
2023-09-01	January	2023	eneral Medici	Self	32	16 to 39	Female	Rural	Winter				URC				Yes	No	Generic	Azithromycin	Watch	Macrolides
2023-10-01	January	2023	Gynaecology	Self	23	16 to 39	Female	Rural	Winter	Itching	White discharge						Yes	No	Generic	Ofloxacin + Ornidazole	Watch	Second generation Fluoro
2023-10-01	January	2023	Gynaecology	Self	39	16 to 39	Male	Rural	Winter	Itching	Burning sensation	Pain in genitals					Yes	No	Generic	Ofloxacin + Ornidazole	Watch	Second generation Fluoro
2023-10-01	January	2023	MD Medicine	Self	48	40 to 65	Male	Urban	Winter	Cough (non productive)					15		Yes	No	Generic	Amoxyclav	Access	Beta-lactam/Penicil
2023-10-01	January	2023	Paediatrics	Other	8	6 to 15	Male	Rural	Winter	Abdominal pain			Tonsillitis	UTI??			Yes	No	Generic	Amoxyclav	Access	Beta-lactam/Penicil
2023-12-01	January	2023	Dental	Self	22	16 to 39	Male	Rural	Winter	Caries	Tooth pain						Yes	No	Generic	Ofloxacin + Ornidazole	Watch	Second generation Fluoro
2023-12-01	January	2023	Gynaecology	Self	30	16 to 39	Female	Rural	Winter	Antenatal	White discharge				2		Yes	No	Generic	Nitrofurantoin	Access	Nitrofurans
2023-12-01	January	2023	Gynaecology	Self	28	16 to 39	Female	Rural	Winter	Antenatal	ful/ Burning micturition						Yes	No	Generic	Nitrofurantoin	Access	Nitrofurans
2023/16/01	January	2023	Dermatology	Self	46	40 to 65	Male	Urban	Winter				Furunculosis	Cellulitis			Yes	No	Generic	Amoxyclav	Access	Beta-lactam/Penicil
2023/16/01	January	2023	MD Medicine	Self	17	16 to 39	Female	Rural	Winter	Increased frequency of micturition			UTI				Yes	No	Generic	Amoxyclav	Access	Beta-lactam/Penicil
2023/16/01	January	2023	Gynaecology	Other	20	16 to 39	Female	Urban	Winter								Yes	No	Generic	Nitrofurantoin	Access	Nitrofurans

Figure S1. The Excel template displaying different data fields extracted from the prescription slips.

Table S1. Patterns of Antimicrobial Prescription by Diagnosis, Classification, and Spectrum.

S No.	Diagnosis/ Sign/ Symptom	Broad categorization	Top antimicrobials prescribed for it	Class of antimicrobial	Broad and Narrow spectrum
1.	Caries	Dental condition	Ofloxacin+ornidazole	2nd generation Fluoroquinolones	Broad spectrum
			Amoxicillin	Aminopenicillins	Broad spectrum
			Amoxicillin- Metronidazole	Aminopenicillins + 2nd generation Fluoroquinolones	Broad + Narrow spectrum
2.	ARI	Respiratory condition	Amoxicillin + clavulanic acid	Aminopenicillins + Beta- lactamase	Broad spectrum
			Cefixime	3rd generation Cephalosporins	Broad spectrum
			Azithromycin	Macrolides	Broad spectrum
3.	UTI	Urogenital condition	Cefixime	3rd generation Cephalosporins	Broad spectrum
			Nitrofurantoin	Nitrofuran	Broad spectrum
			Amoxicillin + clavulanic acid	Aminopenicillins + beta lactamase inhibitor	Broad spectrum
4.	Tonsillitis	Otolaryngologic	Amoxicillin + clavulanic acid	Aminopenicillins + beta lactamase inhibitor	Broad spectrum

		al condition	Azithromycin	1st generation Macrolides	Broad spectrum
			Cefixime	3rd generation Cephalosporins	Broad spectrum
5.	Acne / Acne vulgaris	Respiratory condition	Azithromycin	1st generation Macrolides	Broad spectrum
			Doxycycline	1st generation Tetracycline	Broad spectrum
6.	Cellulitis	Skin condition	Amoxicillin + clavulanic acid	Aminopenicillins + beta- lactamase inhibitor	Broad spectrum
			Cefixime	3rd generation Cephalosporins	Broad spectrum
7.	Fever	Respiratory condition	Azithromycin	1st generation Macrolides	Broad spectrum
			Cefixime	3rd generation Cephalosporins	Broad spectrum
			Doxycycline	1st generation Tetracyclines	Broad spectrum
8.	Diarrhea	Gastroenteritis	Ciprofloxacin	2nd generation Fluoroquinolones	Broad spectrum
			Ofloxacin + ornidazole	2nd generation Fluoroquinolones	Broad spectrum

[redacted]

10.[illegible]

HTN (E).

c/o B/L palm pain
c/o burning micturation
x10 c/o fever.

Adv.

T. Nitrofurantoin 100mg (BD)
Cap. Dexlansoprazole 30/60mg OD [illegible]
Syp. Alkalizer 5ml (TDS) x a week
T. Aceclofenac 100mg (BD)

Adv.
Urine R/E (108)
Urine C/S (111)
CBC test
RA factor (108)
ESR

15.1.20[illegible]

[redacted]

C/o burning micturition

Rx

- T. Nitrofurantoin 100mg BD x 5d
- Syp. Alkalizer 1TSF – BD x 5d
  (c̄ full glass of water)
- T. Dicyclomine 10mg BD x 5d.
- R/A Inv.

Adv

- Urine-R/M
- Urine C/S
- HbA1c
- RBS

[redacted]

C/O ↑ed for micturition
: 1yr

Adv
Urine RE / ME — 10-15 pus cells
Alb+
FBS - 96
HbA1c - 5.8

Rx:
- R/Adv

13/1/25

Adv
Urine c/s - [illegible]
USG KUB

- Tab Nitrofurantoin 100 BD x 1wk
- Syp Citralka 5ml TDS x 5d.
- Plenty of fluids

27th January, 2023

Dear Gitika,

The HISP India REAC (Research Ethics Advisory Committee) reviewed and discussed the proposal entitled **"Digital Equity in Antimicrobial (AMR) policy and practice, which aims to build equity in Digital Global Health"** submitted by Dr. Gitika Arora, in meeting held January 10th 2023.

Decision: The committee approves the research work to be conducted in its present form, with the direction that :

1. Data will be collected only after receiving letter of approval from the HISP India REAC as per submitted undertaking by the applicant.
2. The HISP India REAC needs to be adequately informed on the following:

- Interviewee information sheet and consent forms duly filled and signed.
- Any serious adverse event occurring during the study.
- Final report of the study.

The HISP India REAC must be informed, and permissions must sought before any changes in the protocol, information sheet, informed consent, site of study or investigator.

It must be ensured that there shall be no financial burden on the research participants because of participation in the study and no direct cash incentives are given to the participants.

Please note that members of the HISP India REAC have the right to monitor the study in any phase.

Warm Regards,

A Jain

Society for Health Information Systems Programmes ★ HISP India ★

Member Secretary
HISP India Research Ethics Advisory Committee
